# Photo editing and the risk of anorexia nervosa among children and adolescents

**DOI:** 10.1186/s13052-024-01803-w

**Published:** 2024-11-04

**Authors:** Elena Bozzola, Elena Scarpato, Cinthia Caruso, Rocco Russo, Tommaso Aversa, Rino Agostiniani

**Affiliations:** 1 Pediatric Unit, Bambino Gesù Children’s Hospital, IRCCS, Rome, Italy; 2 The Italian Pediatric Society, Rome, Italy

**Keywords:** Photomanipulation, Anorexia nervosa, Children, Adolescents, Social media

## Abstract

Filters and photoediting are widely used to transform or alter photos, mainly selfies, before sharing with friends or on social networks. In adult population there is a strong evidence of the potential risks of this behaviuor. Aim of the present work is to revise international literature exploring the correlation between photo manipulation and anorexia nervosa among children and adolescents. International literature focusing on photo manipulation and anorexia nervosa has been examined, according to the PRISMA Extension guidelines for Scoping Reviews using the following strategies: “Photomanipulation” Filters: English, Child: 6–12 years, Adolescent: 13–18 years, from 2000–2024 Pubmed Search: ((“Photography“[Mesh]) AND “Anorexia Nervosa“[Mesh]) AND “Anorexia Nervosa“[Majr] Filters: Adolescent: 13–18 years, Child: 6–12 years, from 2000–2024. According to the literature review strategy, only few and limited evidences are available for the pediatric population. As well as in adults, there is an increased risk for eating disorders in adolescents regularly sharing selfies and practicing photo manipulation. New social media and online chat may be associated with lower personal weight satisfaction, higher drive for thinness, and eating disorder symptoms. The Italian Pediatric Society Communication Group suggests to increase the awareness on the potential risks of photo manipulation among children and adolescents, suggesting the plan of more studies target to this population to gain evidence specifically, social campaigns and school education. Finally, the use of technology should be included as part of routine pediatric control visit, especially in the pre-adolescence period.

## Background

Photo manipulation is defined as the process of transforming or altering a photograph - especially selfies - by using editing programs, software or apps before sharing it, in order to achieve desired results [1]. This may range from basic retouching, like light adjustments or cropping, to more complex processes. Editing tools and apps directly integrated into smartphones make photo manipulation extremely easy, allowing to remove imperfections, add filters, correct brightness, etc. While photo manipulation offers exciting prospects for creative expression and innovation, its power to distort reality raises issues regarding societal perceptions and reality distortion. In fact, by using editing tools and image filters built into various apps, users can make themselves look differently from how they really are, creating unrealistic profiles. The main risk associated to photo manipulation is indeed perpetuating unrealistic beauty standards. There are even photo editing applications that use artificial intelligence to fully alter the physical aspect. In this case, technology choses which physical features require modification, potentially decreasing self-esteem. In fact, people may discover flaws in themselves that not have noted before [2]. Over the past few years, social media have increasingly become an integral part of our lives, often representing a showcase through which displaying a “perfect and happy” version life, propagating appearance-focused contents, thus encouraging creators to modify their photos to impress others. Edited photos are frequently shared using social media and findings from studies involving adults support the correlation between social media posting of edited selfies and worse women’s perception of body image and satisfaction with body size. Existing literature has proved that women that are more prone to social media photo editing report greater disordered eating attitudes and behaviors [3]. Moreover, Facebook behaviors may also be associated to bulimic symptoms and overeating episodes [4]. In addition, social media contents related to appearance can have an impact on body image perception and also on cosmetic surgery intention, since body image dissatisfaction may encourage subjects to search for remedies to their disorder, such as cosmetic procedures or surgery. As demonstrated by Herman et al. in a study involving 470 Instagram users aged 18–25 years, passive (e.g., following influencers) and active (e.g., frequency of using Instagram filters) use of highly visual social media are associated with an increased acceptance and normalization of cosmetic procedures, as well as the hypothetical intention to undergo them [5]. Moreover, body dissatisfaction predicts the development of eating disorder symptoms in young adults, representing a strong risk factor for those disorders [6]. 

Aim of the present work is to revise international literature exploring the correlation between photo manipulation and and anorexia nervosa among children and adolescents.

## Materials and methods

International literature focusing on photo manipulation and anorexia nervosa has been examined.

This scoping review has been performed according to the PRISMA Extension guidelines for Scoping Reviews (Jones, M.E. LibGuides: Creating a PRISMA Flow Diagram: PRISMA. 2020. Available online: https://guides.lib.unc.edu/prisma/step-by-step).

An electronic search was undertaken on PubMed database on 24th April 2024 using the following strategies: “Photomanipulation” Filters: English, Child: 6–12 years, Adolescent: 13–18 years, from 2000–2024 Pubmed Search: ((“Photography“[Mesh]) AND “Anorexia Nervosa“[Mesh]) AND “Anorexia Nervosa“[Majr] Filters: Adolescent: 13–18 years, Child: 6–12 years, from 2000–2024.

The research results were downloaded from PubMed and then uploaded on the web application “Rayyan” [Ouzzani, M.; Hammady, H.; Fedorowicz, Z.; Elmagarmid, A. Ray-yan-a Web and Mobile App for Systematic Reviews. Syst. Rev. 2016, 5, 210], a website used to screen and analyze articles, specific for writing reviews.

As the first step, duplicates were identified by the web application, Rayyan. After that, authors evaluated duplicates excluding copies. To limit errors and bias, two authors independently screened titles and abstracts and identified articles irrelevant to the review.

Exclusion criteria were:

- Reports including adults, without age distinction.
- Reports dealing with other themes different from the topic of investigation.

Afterward, full texts were retrieved and assessed for eligibility by the screening authors. Finally, following PRISMA guidelines, references not included in the original search but relevant to the review were examined. Disagreements regarding inclusion/exclusion were settled through discussion between the researchers.

## Results

The search on the selected database produced n°98 articles and reviews. Documents were analyzed to confirm their relevance and eligibility. No duplications were identified by researchers.

According to PRISMA guidelines, of the identified items, all abstracts were analyzed, 91 records were excluded as irrelevant, and 5 records were excluded as based on adult population.

In conclusion, 2 records were included in the revision [1, 3]. 

**Fig. 1:**
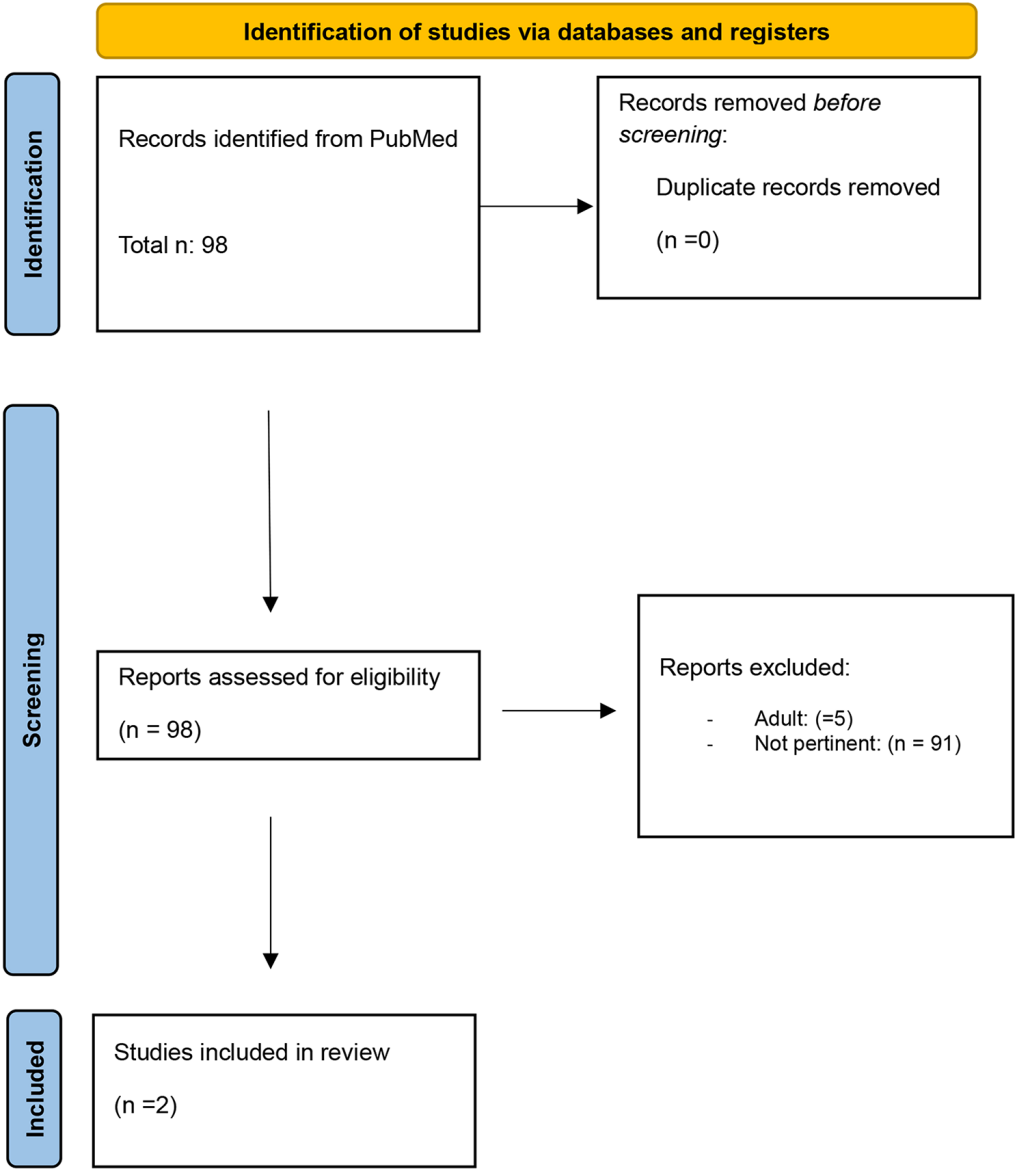
Flow chart of the selection progress

Figure 1 presents the flow chart of the selection process, adapted from PRISMA guidelines. (Fig. 1)

In details, Lonergan et al. found an association between appearance-related social media behaviors and eating disorders in a sample of Australian adolescents. Moreover, investigating the effects of posting selfies, photo investment and photo manipulation, they found a higher risk for eating disorders, mainly among males [3]. Data form Lonergan et al. were in line with former findings from McLean et al., that explored the link between social media use, body perception and eating concerns in grade seven girls, with a mean age of 13 years. Specifically, the Authors found that girls regularly sharing selfies on social media tended to over-evaluate shape, weight, and body dissatisfaction, compared to those not sharing selfies. Moreover, among girls who shared selfies, higher engagement in manipulation was associated with greater body-related and eating concerns [1]. Table 1 summarizes the evidence and summarize the characteristics of studies. (Table 1)

**Table 1:** Photo editing and the risk of anorexia

Author	Country	Participants	Age	Main findings
Mc Lean SA, 2015 [1]	Australia	101 females Provenience: 91%Australia, 9% East Asia, Europe, and New Zealand	Mean age 13.3 years	Girls who regularly shared self-images on social media reported significantly higher overvaluation of shape and weight, body dissatisfaction, dietary restraint, and internalization of the thin ideal. Those with a higher engagement in photo manipulation manifested body-related and eating concerns
Lonergan AR, 2020 [3]	Australia	4209 (53.15% female) Provenience: 89.2% Australia, 5.7% Asia, 2.2% Europe, 1.3% Oceania, 0.9% Africa, 0.6% America	Age range: 12–18 years for boys, 11–19 years for girls	Social media behaviours, including photo investment, and photo manipulation correlate to an increased risk of developing eating disorders.

## Discussion

Eating disorders are defined as a group of diseases characterized by altered eating behaviors and body image concerns. In adulthood, evidence suggests an association between photo manipulation, body dissatisfaction, and reduced self-esteem. It has also been suggested that photo manipulation could reinforce risks of body shame, depression, and eating disorders [7, 8]. Thus, body image perception represents a key risk factor for the development of eating disorders [9, 10]. Posting edited selfies is common between minors, with more than 90% of the teens that use social media posting photos of themselves Nevertheless, scientific evidence on the consequences of photo manipulation lacks. Examining the literature, a relationship between disordered eating and body image has been found in adolescent girls with a greater effort in choosing a selfie and monitoring likes/comments. So, photo manipulation with digital editing of a selfie, seems to be linked not only to decreased personal perception, but also to eating concerns. Literature revision provide insight in an association between appearance-related social media behaviors and eating disorders in minors [1, 3]. 

Photo editing exposes females to greater peer scrutiny of appearance and competition, which, from an evolutionary perspective, has been proposed to increase body dissatisfaction and body concerns. Taking, sharing and comparing selfies through social media, may heighten appearance focus and increase internalization of appearance ideals, contributing to body dissatisfaction. In addition, monitoring of likes/comments on the uploaded selfies may represent an online manifestation of reassurance-seeking [11]. 

New social media and online chat, including Facebook, Instagram, Snapchat, and TikTok exposure may be associated with lower personal weight satisfaction, higher drive for thinness, and eating disorder symptoms. Studies revealed that time spent on the Internet is significantly related to internalization of the thin ideal, body surveillance, and drive for thinness. In particular, Facebook users scored significantly higher on all body image concern measures than non-users [12]. The more vulnerable the subjects are, the greater the risk is. Young adolescent girls with high levels of body-related and eating concerns might engage in social media activities manipulating selfies to present an ideal appearance when sharing images. In the paper by McLean et al., 20 out of 74 photos had been manipulated. In the selfie-sharing group, higher scores for selfie manipulation were associated with higher scores for body-related and eating concerns [1]. 

A topic of concern is that social media use among adolescents is increasing, despite the fact that social media access for minors is restricted by age in many countries. In Italy, many platforms, including TikTok, Instagram, Facebook, and YouTube, have age restrictions. However, without a strict parental control, minors can easily access social media platform and download the applications.

Even though social media may help adolescents to build social networks, connect, chat and stay in touch with old and new friends, their use may also be linked to potential harms and unhealthy mental effects, following exposure to inappropriate videos and images, especially in vulnerable categories like adolescents.

A limitation of the study is the few available data connected to few evidences on the pediatric population. Further studies may add important information and improve awareness of this phenomena. Even if our systematic has been based by a detailed strategy adopting PRISMA guideline, we have to declare the risk of selection bias as we limited our review to Pubmed database as is the most extensively used database and search engine in the biomedical and healthcare fields, contain more than 37 million citations and abstracts of biomedical literature. We excluded other datasets, unpublished papers, clinical trial registries, regulatory agency websites, and conference abstracts. Another bias may regard contributing reports focusing on the provenience of the examined population, mainly Australian, and on the different inclusion criteria used by the authors which contribute heterogeneity to the results. Finally, as we got just two findings, no sensitivity analyses have been adopted to compare the reports.

## Conclusion and call to action

Although the etiology of anorexia nervosa is not defined and likely multifactorial, exposure to social media may be an important contributor to the onset or worsening of symptoms [13]. Editing tools and image filters may create unrealistic beauty standards and increase the body dissatisfaction in vulnerable adolescents [1, 3]. As demonstrated by a previous survey of the Italian Pediatric Society, eating disorders as well as neuropsychological impairments are increasing among the pediatric population [14]. In order to prevent the risk of a further increase of this group of diseases, the Italian Pediatric Society Communication Group suggests the following action:

- Conducing specific studies on the effect of photo manipulation and sharing edited photo among children and adolescents, to gain evidence specifically target to this population;
- Launch of social campaigns to increase the awareness on photo manipulation risks, in order to prevent neuropsychological effects on minors;
- Providing school education on the use of media devices and social media, underlying risks and benefits of their use and strategies to prevent negative effects;
- Considering the education for a conscious use of technology as part of routine pediatric control visit, especially in the pre-adolescence period;
- Improving parents’ awareness on filters and photo editing possible consequences on adolescents’ health.

## Data Availability

Not applicable.

## References

[1] McLean SA, Paxton SJ, Wertheim EH, Masters J. Photoshopping the selfie: self photo editing and photo investment are associated with body dissatisfaction in adolescent girls. Int J Eat Disord. 2015;48(8):1132–40. 10.1002/eat.22449. Epub 2015 Aug 27. PMID: 26311205. 26311205 10.1002/eat.22449

[2] Lavrence C, Cambre C. (2020). Do I look like my selfie? Filters and the digital-forensic gaze.Social Media + Society, 6(4).

[3] Lonergan AR, Bussey K, Fardouly J, Griffiths S, Murray SB, Hay P, Mond J, Trompeter N, Mitchison D. Protect me from my selfie: examining the association between photo-based social media behaviors and self-reported eating disorders in adolescence. Int J Eat Disord. 2020;53(5):485–96. 10.1002/eat.23256. Epub 2020 Apr 7. PMID: 32259344. 32259344 10.1002/eat.23256

[4] Smith AR, Hames JL, Joiner TE. Status update: Maladaptive Facebook usage predicts increases in body dissatisfaction and bulimic symptoms. J Affect Disord. 2013;149(1):235–40. 10.1016/j.jad.2013.01.032. 23453676 10.1016/j.jad.2013.01.032

[5] McGovern O, Collins R, Dunne S. The associations between photo-editing and body concerns among females: a systematic review. Body Image. 2022;43:504–17. 36371869 10.1016/j.bodyim.2022.10.013

[6] Stice E, Marti CN, Durant S. Risk factors for onset of eating disorders: evidence of multiple risk pathways from an 8-year prospective study. Behav Res Ther. 2011;49(10):622–7. 21764035 10.1016/j.brat.2011.06.009 PMC4007152

[7] Lamp SJ, Cugle A, Silverman AL, Thomas MT, Liss M, Erchull MJ. Picture perfect: the relationship between selfie behaviors, self-objectification, and depressive symptoms. Sex Roles. 2019;81(11):704–12.

[8] Fredrickson BL, Roberts TA. Objectification theory: toward understanding women’s lived experiences and mental health risks. Psychol Women Q. 1997;21(2):173–206.

[9] Fardouly J, Pinkus RT, Vartanian LR. The impact of appearance comparisons made through social media, traditional media, and in person in women’s everyday lives. Body Image. 2017;20:31–9. 27907812 10.1016/j.bodyim.2016.11.002

[10] Culbert KM, Racine SE, Klump KLJ, Research Review. What we have learned about the causes of eating disorders - a synthesis of sociocultural, psychological, and biological research. Child Psychol Psychiatry. 2015;56(11):1141–64. 10.1111/jcpp.12441 26095891

[11] Chua THH, Chang L. Follow me and like my beautiful selfies: Singapore teenage girls’ engagement in self-presentation and peer comparison on social media. Comput Hum Behav. 2016;55:190–7.

[12] Tiggemann M, Slater A, NetGirls. The internet, Facebook, and body image concern in adolescent girls. Int J Eat Disord. 2013;46:630–3. 23712456 10.1002/eat.22141

[13] Sharma A, Vidal C. J A scoping literature review of the associations between highly visual social media use and eating disorders and disordered eating: a changing landscape. Eat Disord. 2023;11(1):170. 10.1186/s40337-023-00898-6 PMC10521472 37752611

[14] Bozzola E, Ferrara P, Spina G, Villani A, Roversi M, Raponi M, Corsello G, Staiano A. Italian Pediatric COVID-19 Board.The pandemic within the pandemic: the surge of neuropsychological disorders in Italian children during the COVID-19 era. Ital J Pediatr. 2022;48(1):126. 35897109 10.1186/s13052-022-01324-4 PMC9326438

